# Ancestral Resurrection and Directed Evolution of Fungal Mesozoic Laccases

**DOI:** 10.1128/AEM.00778-20

**Published:** 2020-07-02

**Authors:** Bernardo J. Gomez-Fernandez, Valeria A. Risso, Andres Rueda, Jose M. Sanchez-Ruiz, Miguel Alcalde

**Affiliations:** a Department of Biocatalysis, Institute of Catalysis and Petrochemistry, CSIC, Madrid, Spain; b EvoEnzyme, S.L., Madrid, Spain; c Departamento de Química Física, Facultad de Ciencias, Universidad de Granada, Granada, Spain; d INRS–Institut Armand-Frappier, Université du Québec, Laval, Quebec, Canada; University of Toronto

**Keywords:** ancestral reconstruction, directed evolution, laccase

## Abstract

Ancestral sequence reconstruction and resurrection provides useful information for protein engineering, yet its alliance with directed evolution has been little explored. In this study, we have resurrected several ancestral nodes of fungal laccases dating back ∼500 to 250 million years. Unlike modern laccases, the resurrected Mesozoic laccases were readily secreted by yeast, with similar kinetic parameters, a broader stability, and distinct pH activity profiles. The resurrected Agaricomycetes laccase carried 136 ancestral mutations, a molecular testimony to its origin, and it was subjected to directed evolution in order to improve the rate of 1,3-cyclopentanedione oxidation, a β–diketone initiator commonly used in vinyl polymerization reactions.

**IMPORTANCE** The broad variety of biotechnological uses of fungal laccases is beyond doubt (food, textiles, pulp and paper, pharma, biofuels, cosmetics, and bioremediation), and protein engineering (in particular, directed evolution) has become the key driver for adaptation of these enzymes to harsh industrial conditions. Usually, the first requirement for directed laccase evolution is heterologous expression, which presents an important hurdle and often a time-consuming process. In this work, we resurrected a fungal Mesozoic laccase node which showed strikingly high heterologous expression and pH stability. As a proof of concept that the ancestral laccase is a suitable blueprint for engineering, we performed a quick directed evolution campaign geared to the oxidation of the β-diketone 1,3-cyclopentanedione, a poor laccase substrate that is used in the polymerization of vinyl monomers.

## Abstract (precis)

The broad variety of biotechnological uses of fungal laccases is beyond doubt (food, textiles, pulp and paper, pharma, biofuels, cosmetics, and bioremediation), and protein engineering (in particular, directed evolution) has become the key driver for adaptation of these enzymes to harsh industrial conditions. Usually, the first requirement for directed laccase evolution is heterologous expression, which presents an important hurdle and often a time-consuming process. In this work, we resurrected a fungal Mesozoic laccase node which showed strikingly high heterologous expression and pH stability. As a proof of concept that the ancestral laccase is a suitable blueprint for engineering, we performed a quick directed evolution campaign geared to the oxidation of the β-diketone 1,3-cyclopentanedione, a poor laccase substrate that is used in the polymerization of vinyl monomers.

## INTRODUCTION

Although more than 50 years have passed since Pauling and Zuckerkandl declared that it would be possible to infer the sequence of ancestral enzymes in the near future (1), their prediction does finally appear to have become a reality (2–4). Current advances in molecular and computational biology, along with the constant drop in the price of DNA synthesis, have made ancestral enzymes more accessible to laboratories around the world. Indeed, through ancestral sequence reconstruction and resurrection (i.e., the functional expression of inferred ancestral nodes in modern microbes), “*paleoenzymologists*” are bringing back protein sequences from long-extinct organisms in an attempt to scrutinize the events of natural molecular evolution. From a strict protein engineering point of view, resurrected enzymes may exhibit a range of appealing biochemical traits, such as stability, promiscuity, and heterologous expression, all of which characteristics may be interesting to instill in modern enzymes (5–12). Under the assumption that ancient microorganisms could not afford a broad repertoire of specialist enzymes (due to the high associated metabolic cost), the majority of the tasks within ancestral microorganisms were performed by just a few generalist biocatalysts, which in turn had to adapt to the harsh environments of ancient earth (particularly those in the Precambrian period) (13–16). As such, it seems plausible that ancestral enzymes might be suitable workhorses for directed evolution enterprises aimed at reviving more promiscuous activity or promoting the evolution of enzymes toward functions apparently never explored in nature (6, 7, 16–19). We recently showcased the first proof of concept where directed evolution and ancestral resurrection were aligned to improve the expression, mutational tolerance, and stability of a Precambrian RubisCO. These studies open novel engineering opportunities that could also be transferred to other enzyme systems (6).

Laccases are multicopper oxidases that act on a wide variety of compounds, using oxygen from the air as the final electron acceptor and producing water as the only by-product (20–22). Their catalytic cycle involves one Cu atom at the T1 site for the oxidation of the reducing substrate and the T2/T3 trinuclear Cu cluster for the reduction of molecular oxygen. High-redox-potential laccases secreted by white rot fungi are in high demand due to their superior catalytic performance and versatility (23–25). However, fungal laccases need to be adapted to the harsh demands of industry, such that their heterologous expression, activity, and/or stability at extreme temperatures or pH values can be included in the priorities of the engineering wish list (26–32).

In this study, we have reconstructed and resurrected several ancestral nodes of fungal laccases dating back ∼500 to 250 million years (estimated). Biochemical benchmarking with a modern counterpart (32) revealed improved heterologous expression in yeast, coupled to a broader pH activity and stability profile. To prove the versatility of ancestral laccases, the Mesozoic node was subjected to structure-guided evolution toward the oxidation of 1,3-cyclopentanedione, a β–diketone initiator used for vinyl polymerization reactions.

## RESULTS AND DISCUSSION

### Reconstruction and resurrection of ancestral laccases.

**FIG 1:**
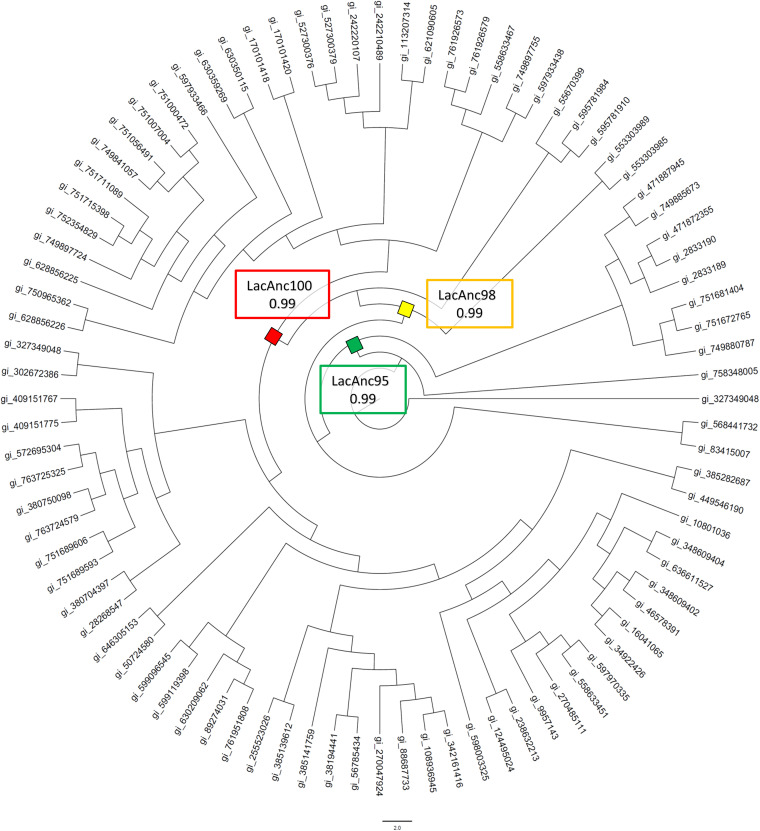
Phylogenetic tree generated from the amino acid sequences of 87 different laccases sequences. The nodes whose sequences were selected for resurrection are depicted as squares. Green square, LacAnc95 the common ancestor of some Basidiomycota; yellow square, LacAnc98, the common ancestor of Agaricomycotina; red square, LacAnc100, the common ancestor of Agaricomycetes. The TimeTree of Life (available at http://www.timetree.org/) was employed to locate the ancestral nodes at the phylogenetic level. The tree was designed with FigTree software v.1.4.1 (available at http://tree.bio.ed.ac.uk/software/figtree/).

**FIG 2:**
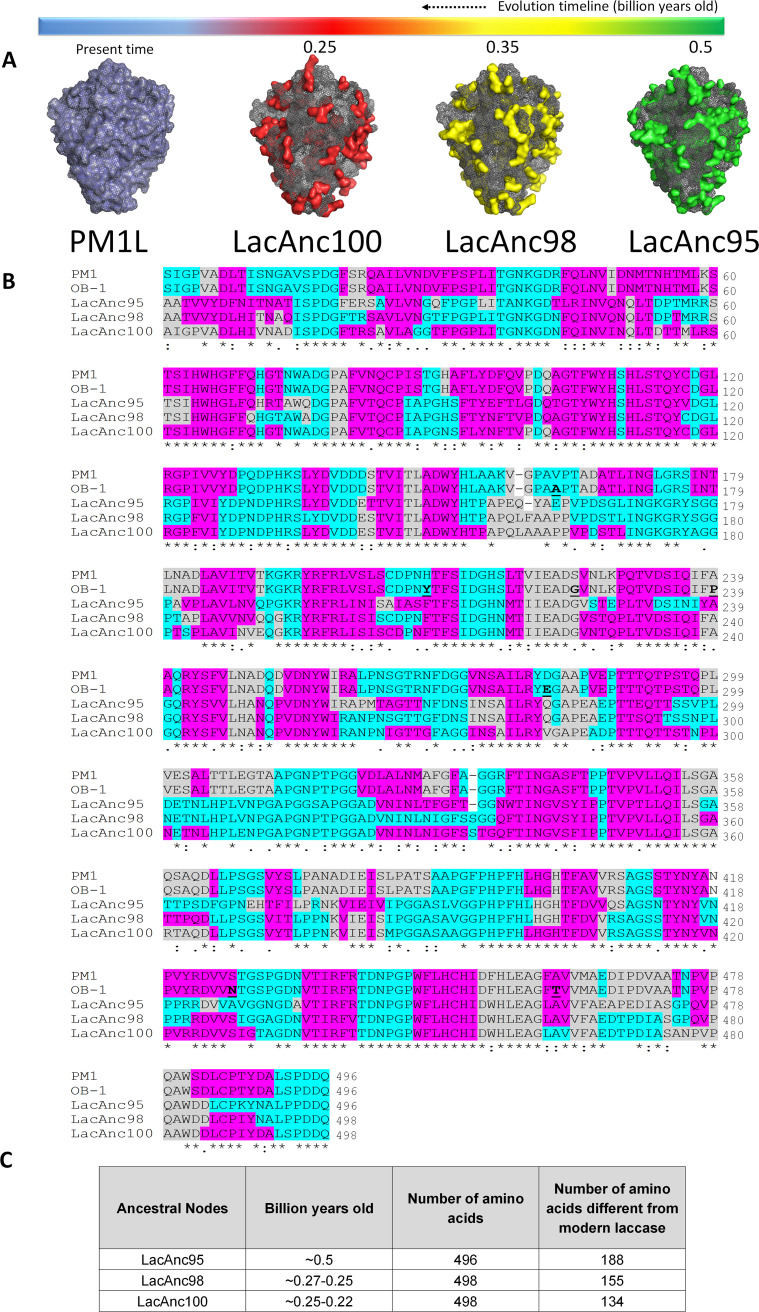
Comparison between modern and ancestral/resurrected laccases. (A) Amino acid differences (in surface mode) in LacAnc95 (green), LacAnc98 (yellow), and LacAnc100 (red) relative to PM1L. (B) Multiple sequence alignment of PM1L, OB-1, and ancestral laccases. Expected motifs are highlighted in colors (pink, beta sheets; gray, alpha helices; blue, loops). Mutations found during the directed evolution of PM1L to OB-1 are highlighted in bold and underlined. An asterisk indicates positions with a fully conserved residue, a colon indicates conservation between groups of strongly similar properties, and a period indicates conservation between groups of weakly similar properties. (C) Number of amino acids different from PM1L and billion years old between the ancestral nodes. The multiple sequence alignment was performed with Clustal Omega V1.2.4, available at http://www.ebi.ac.uk/Tools/msa/clustalo/. The structures were modeled using the PDB code 5ANH and the Phyre2 server (Protein Homology/analogY Recognition Engine V 2.0), available at www.sbg.bio.ic.ac.uk/phyre2.

**(i) Ancestral reconstruction and sequence analysis.** Using the PM1 high-redox-potential laccase (PM1L) from basidiomycete as a query sequence (32), we reconstructed three ancestral laccase nodes: LacAnc95, LacAnc98, and LacAnc100, Fig. 1. The TimeTree of Life was employed to locate the ancestral nodes at the phylogenetic level, dating them back 500 to 252 million years (at the beginning of the Phanerozoic eon), within the appearance of the common ancestor of some Basidiomycota, in the early Cambrian period (LacAnc95), and the common ancestors of Agaricomycotina and Agaricomycetes, in the Mesozoic era (LacAnc98 and LacAnc100) (33) (Fig. 1 and 2; Fig. S1 and S2 in the supplemental material). Indeed, the prelude of the Mesozoic era, the Permian-Triassic extinction, spawned environmental stress and disturbance, factors that accelerate the genetic response for living organisms to survive (34), so that new biochemical traits arose. Given the large difference in the protein sequence between modern and ancestral nodes (roughly 40% amino acid difference between LacAnc95 and modern PM1L) (Fig. 2C), we made a multiple-sequence alignment (MSA) to reveal the number of positions in the resurrected enzymes not present in any other laccase registered to date. After analyzing over 100 basidiomycete laccases from the NCBI database, no positive matches were found. These results are not unexpected since the calculations for the probabilities for every single amino acid are strongly affected by the number of times that each residue is present in the sequences employed in the reconstruction. When we made the biochemical characterization of the resurrected ancestral nodes (see below), we compared them to the OB-1 variant, the product of eight generations of directed evolution performed on the PM1L to enhance secretion, activity, and stability (32). OB-1 carries the following mutations in the mature protein: V162A-H208Y-S224G-A239P-D281E-S426N-A461T. To discern whether any of these substitutions that increased activity and secretion were ancestral mutations, we aligned the OB-1 laccase sequence with the reconstructed nodes (Fig. 2B). The S224G substitution was the only ancestral mutation found in the OB-1 variant, as a Gly residue at the same position appeared in the three ancestral laccase nodes, whereas a Ser was in this place in modern PM1L. The effect of this ancestral mutation on the total activity (the product of specific activity and secretion) was indeed dramatic, with an improvement of 7-fold over PM1L (32). In contrast, A239P, S426N, and A461T substitutions were “suppressed-ancestral” mutations because the original residues were mostly present in the ancestral nodes as well as preserved in PM1L (Fig. 2B). The remaining substitutions could not be assigned as ancestral mutations, which addresses that the improved secretion and activity of OB-1 were not directly connected to ancestral mutations, with the notable exception of S224G. It is also worth noting that in a recent work we applied an in-house consensus mutagenesis method to insert 18 ancestral/consensus mutations in OB-1, some of which promoted a strong effect in thermostability, kinetic values, and secretion (35). As such, these results open a venue to combine consensus mutagenesis and ancestral resurrection aimed at engineering more robust and efficient fungal laccases.

**(ii) Resurrection of ancestral nodes.** The three ancestral nodes were cloned in Saccharomyces cerevisiae, the most common host for directed evolution of fungal laccases (31), and laboratory evolved and/or chimeric versions of the α-factor prepro-leader from S. cerevisiae (α^PM1^, α^PcL^, pre-α-prokiller) were attached to each node to enhance their secretion, which was then evaluated (Fig. S3) (32, 36–38). Regardless of the node, the highest secretion levels in microtiter fermentations (cultures in 96-well plates used for laboratory evolution) were obtained using the α^PcL^ signal sequence, which is in good agreement with our previous observations (36). To optimize the culture conditions further, the supernatant from microtiter fermentations of all constructs was screened. As a result, and when assessed with a supply of ethanol at 30°C, those expressing LacAnc98 and LacAnc100 fusions attached to α^PcL^ displayed the highest activity in the microtiter plate supernatants (and were readily detectable for a directed evolution campaign) (36). These results agree well with former studies which showed that ethanol may help improve secretion by enhancing cytoplasmic membrane permeability, as well as by generating a stress response related to protein folding and exocytosis, whereas the production of fungal laccases is generally promoted at 30°C in yeast (32, 38). In contrast, LacAnc95 was not expressed in this format, irrespective of the signal peptide or the culture conditions used.

**(iii) Biochemical characterization.** The LacAnc98 and LacAnc100 variants were produced, purified to homogeneity, and characterized biochemically. Unfortunately, we could not compare the ancestral nodes with the wild-type PM1 laccase (PM1L) produced by S. cerevisiae due to its poor secretion in this host (0.035 2,2′-azino-bis[3-ethylbenzothiazoline-6-sulfonic acid] [ABTS] units/liter of supernatant) (32). Instead, we used the evolved OB-1 laccase for benchmarking with the ancestral nodes. LacAnc98 and LacAnc100 had a molecular mass determined by SDS-PAGE of ∼65 kDa, with ∼15% glycosylation (estimated from the deglycosylation pattern), as with evolved OB-1 (32) (Table 1, Fig. S4A). To correctly assess expression, OB-1 had to be fused to α^PcL^, the same prepro-leader attached to the Mesozoic nodes, which proved to be a valuable signal peptide for laccase microtiter expression (36). In this format, the ancestral fusions showed 10-fold higher secretion than the α^PcL^-OB-1 fusion (10 mg/liter versus 1 mg/liter), which is particularly striking in light of the general difficulties found when native fungal laccases are heterologously expressed. Indeed, fungal laccases typically require exhaustive directed evolution to achieve reasonable levels of secretion (32, 36, 38, 39), and our findings show that protein resurrection can bypass laccase expression problems by inferring and expressing a relatively close ancestor.

**TABLE 1:** Biochemical features of wild-type PM1L, evolved OB-1, and resurrected nodes

Feature	PM1L^*a*^	OB-1	LacAnc98	LacAnc100
MW (kDa)^*b*^	56	60	65	65
pI^*b*^	5.4	5.1	5.1	4.8
Glycosylation (%)	6	12	15	15
*t*_1/2_ at 50°C and pH 3.0 (min)	ND	9	52	65
*t*_1/2_ at 70°C and pH 6.0 (min)	ND	25	4	5
Optimum pH for ABTS	2–3	2–3	2–3	2–3
Optimum pH for DMP	4	4	4	5
Optimum pH for K_4_Mo(CN)_8_	ND	2	2	2
Optimum pH for guaiacol	ND	4	5	5

a PM1L from the original basidiomycete PM1. Data were extracted from reference 40. ND, not determined.

b The predictions of pI and molecular weight (MW) were calculated using the ExPASy Compute pI/molecular weight (MW) tool, available at https://web.expasy.org/compute_pi/.

**FIG 3:**
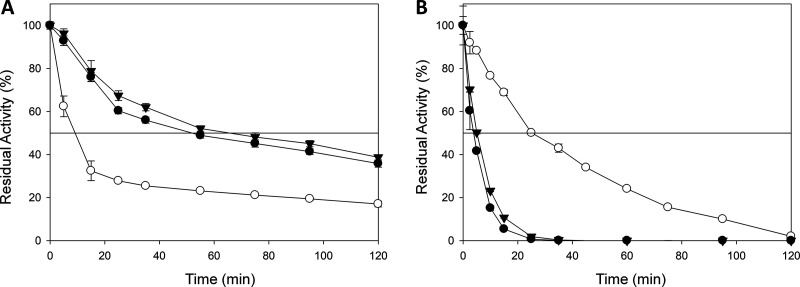
Half-life (*t*_1/2_) of LacAnc98, LacAnc100, and the OB-1 variant in pH 3.0 at 50°C (A) and pH 6.0 at 70°C (B). The solid line shows the residual activity at 50%. White circles, OB-1; black circles, LacAnc98; black triangles, LacAnc100. Each point, including the standard deviation, comes from three independent experiments.

**FIG 4:**
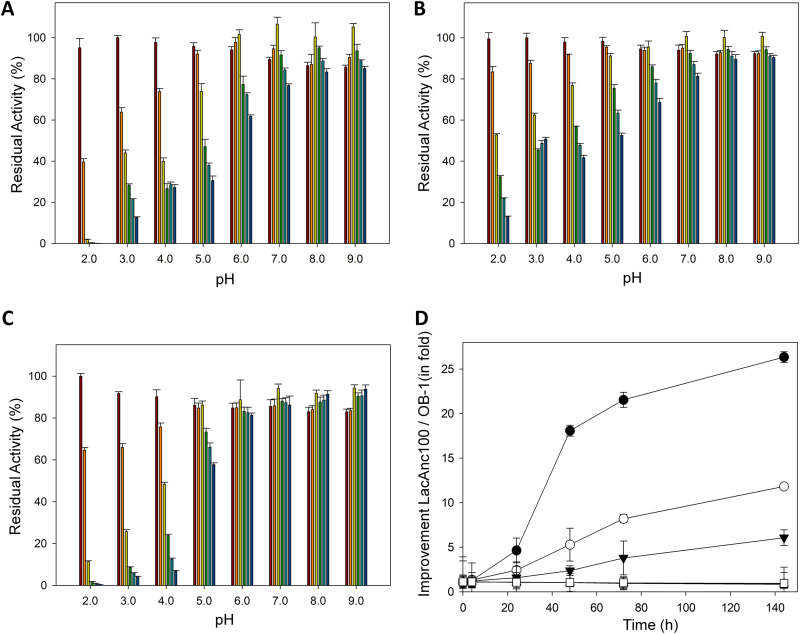
pH stability of LacAnc98 (A), LacAnc100 (B), and the OB-1 variant (C). Laccases were incubated for 0 (brown), 4 (orange), 24 (yellow), 48 (green), 72 (light blue), and 144 (blue) h at different pH values (from 2.0 to 9.0). Laccase activity was normalized to the highest activity value at time zero. Each point and standard deviation is from three independent measurements. (D) Difference in improvement (in fold) of residual activities between LacAnc100 and OB-1 after incubation at different pH values. After the incubation of 0, 4, 24, 48, 72, 96, and 144 h at different pH values (from 2.0 to 7.0), residual activity was measured at room temperature with 1 mM ABTS in 100 mM sodium phosphate/citrate buffer, pH 4.0. Residual activities of LacAnc100 were divided over residual activities of the OB-1 variant at the corresponding pH value to determine the fold of improvement. Black circles, pH 2.0; white circles, pH 3.0; black triangles, pH 4.0; white triangles, pH 5.0; black squares, pH 6.0; white squares, pH 7.0. Each point and the standard deviation come from three independent measurements.

Kinetic stability was assessed by measuring the half-life of inactivation (*t*_1/2_), defined as the time required for the laccase to lose 50% of its initial activity after incubation at a given temperature. When stability was measured at 50°C and pH 3.0, both ancestral nodes outperformed the modern laccase in terms of *t*_1/2_ by roughly 55 min (Fig. 3A, Table 1). In contrast, the *t*_1/2_ at 70°C and pH 6.0 of OB-1 surpassed that of the ancestral laccases by 20 min, indicating a strong effect of pH on overall stability; i.e., thermostability values were dependent on the pH at which the assay was performed (Fig. 3B, Table 1). In light of these results, we studied pH stability in the range of pH 2.0 to 9.0, at room temperature. LacAnc100 showed a general broader stability along the pH range than OB-1 (Fig. 4B and C). Specifically, LacAnc100 retained good stability at acidic pH values and even after long incubation periods (144 h) at pH 2 to 3, conditions in which the residual activity of the modern laccase was hardly measurable. Indeed, this improvement in the stability of LacAnc100 relative to that of OB-1 was enhanced by a gradual increase in acidity (Fig. 4D). LacAnc98 also showed higher stability than OB-1 at acidic pH values, but to a lesser extent than LacAnc100, whereas OB-1 seems to be a little bit more stable at the pH range of 5 to 6 (Fig. 4A to C). Long-term stability at acidic pH values for fungal laccases is an attractive biotechnological feature, as many industrial and environmental processes happen at the pH range of 2 to 5. Generally, differences in activity and stability profiles are the combined consequence of interactions like salt bridges, hydrogen bonds, and hydrophobic contacts. Despite the protein sequence differences between LacAnc100 and modern PM1L (134 different residues), the molecular homology models showed a presumed overall similar structural folding, something that can only be concluded by protein crystallization (Fig. 2). As such, we cannot offer reasonable explanations for the improved acidic pH stability observed in the resurrected laccase, given that the number of contacts involved are difficult to link. However, our results are in good agreement with previous protein resurrection studies in which the stability/activity/expression were improved without substantial structural modifications, suggesting conformational flexibility as the key driving trait of ancient enzymes expressed in modern hosts (6, 7).

**FIG 5:**
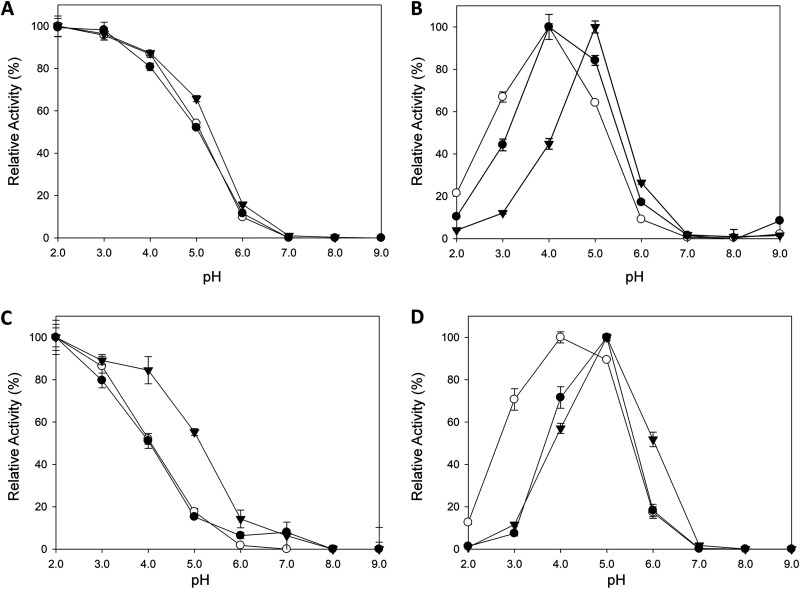
pH activity profiles. White circles, OB-1; black circles, LacAnc98; black triangles, LacAnc100. Activities were measured at room temperature in 100 mM citrate/phosphate/borate buffer at different pH values with 1 mM ABTS (A), 5 mM DMP (B), 5 mM K_4_Mo(CN)_8_ (C), or 50 mM guaiacol (D) as the substrates. Laccase activity was normalized to the optimum activity value and each point, including the standard deviation, comes from three independent experiments.

The pH activity profile was measured for phenolic (2,6-dimethoxyphenol [DMP] and guaiacol) and nonphenolic (2,2′-azino-bis[3-ethylbenzothiazoline-6-sulfonic acid] [ABTS]) compounds, as well as for potassium octacyanomolybdate (K_4_Mo[CN]_8_, an inorganic transition metal complex) (Fig. 5, Table 1). A deviation of LacAnc100 (and to a lesser extent for LacAnc98) toward more alkaline pH was observed for all substrates except for ABTS, with a shift in the optimum pH of activity from 4.0 to 5.0 for the two phenolic substrates.

Steady-state kinetic constants were measured with a panel of laccase substrates (Table 2) and the catalytic efficiency of LacAnc98 was around 1 order of magnitude lower than that of OB-1 for all the substrates assayed. Conversely, LacAnc100 and OB-1 had similar kinetic values except when ABTS was the substrate, for which the *K*_m_ of the ancestral laccase was 10-fold higher than that of the extant one. It is important to note that the kinetic constants of the PM1L produced homologously by the fungus are in the same order as those for OB-1, which was intensively evolved to achieve high activity and secretion in yeast (32). This implies that the activity of the PM1L might be severalfold lower when the enzyme is expressed in S. cerevisiae than when expressed in the original fungus, as also witnessed for other laccases expressed in yeast (39, 40).

**TABLE 2:** Kinetic parameters of evolved OB-1, LacAnc100, and LacAnc98 expressed in yeast and the PM1L from the original basidiomycete PM1^*a*^

Substrate	Kinetic constant	OB-1	PM1L^*b*^	LacAnc100	LacAnc98
ABTS	*K*_m_ (mM)	0.0070 ± 0.0006	0.0081 ± 0.0007	0.060 ± 0.001	0.069 ± 0.002
	*k*_cat_ (s^−1^)	690 ± 20	272 ± 7	1,325.60 ± 0.06	468.44 ± 10
	*k*_cat_*/K*_m_ (mM^−1^ s^−1^)	106,073.8	33,580	23,054.2	6,711.17
DMP	*K*_m_ (mM)	0.15 ± 0.01	0.12 ± 0.01	0.50 ± 0.02	0.42 ± 0.02
	*k*_cat_ (s^−1^)	565 ± 13	153 ± 4	600 ± 10	238 ± 5
	*k*_cat_*/K*_m_ (mM^−1^ s^−1^)	3,842.6	1,093	1,217.3	569.76
Sinapic acid	*K*_m_ (mM)	0.32 ± 0.02	0.048 ± 0.001	0.33 ± 0.07	0.23 ± 0.04
	*k*_cat_ (s^−1^)	741.0 ± 14.1	45 ± 3	520 ± 55	129 ± 11
	*k*_cat_*/K*_m_ (mM^−1^ s^−1^)	2,286.4	923	1,580.0	569.16
Guaiacol	*K*_m_ (mM)	2.6 ± 0.4	1.16 ± 0.03	6.7 ± 0.7	8.7 ± 0.3
	*k*_cat_ (s^−1^)	117 ± 6	65.9 ± 0.5	140 ± 5	37.2 ± 0.5
	*k*_cat_*/K*_m_ (mM^−1^ s^−1^)	44.6	57	21.0	4.28

a Kinetic constants were estimated at room temperature in 100 mM sodium phosphate/citrate buffer pH 4.0 for ABTS and pH 5.0 for DMP, sinapic acid, and guaiacol. All reactions were performed in triplicate.

b Data extracted from reference 40.

In summary, through ancestral resurrection we have obtained a fungal laccase with high levels of secretion when expressed in a heterologous system and with catalytic constants similar to those of the evolved OB-1 variant, yet with enhanced pH stability. These characteristics were obtained without passing through the time-consuming process of iterative random mutation, recombination, and screening of a directed-evolution experiment.

### Directed evolution of the ancestral laccase.

**FIG 6:**
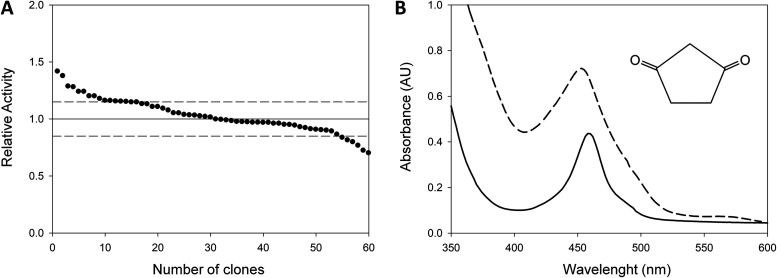
Screening assay design based on 1,3-cyclopentanedione. (A) Activities of the ancestral enzyme plotted in descending order for the 1,3-cyclopentanedione assay. S. cerevisiae cells were transformed with PjRoC30-LacAnc100 and plated on SC plates. Individual colonies were picked and inoculated in a 96-well plate. The activities of the clones were evaluated from fresh supernatant preparations. Dashed lines indicate the coefficient of variation of the assay. (B) UV-VIS spectrum of 1,3-cyclopentanedione in the presence of laccase supernatants. Reaction mixture at *t* = 0 (solid line) and *t* = 40 h (dashed line). Maximum absorbance peak was at 450 nm.

As a proof of concept that LacAnc100 could be modified in new directions, we carried out a preliminary directed laccase evolution experiment toward the oxidation of β-diketones, an engineering goal that has not been accomplished before. β-Diketones are a special class of redox mediators known as initiators that are oxidized by laccases to trigger the polymerization of vinyl monomers, and are themselves incorporated into the final polymeric structure of polystyrene or polyacrylamide (41–45). Although using β-diketones to initiate vinyl polymerization is an attractive alternative for replacing aggressive chemical methods, laccases only oxidize them poorly (42, 46, 47). We thus carefully examined the β-diketone family of compounds and chose 1,3-cyclopentanedione to design a high-throughput screening (HTS) assay for laboratory evolution. The maximal absorbance of this yellow, soluble molecule occurs at 460 nm, whereas upon oxidation by laccase it becomes orange and shifts its absorbance to 450 nm. The HTS assay was validated by establishing the linearity and coefficient of variance (15%) using fresh supernatants produced in microcultures (Fig. 6). Two consecutive rescreenings were performed to rule out false positives.

**FIG 7:**
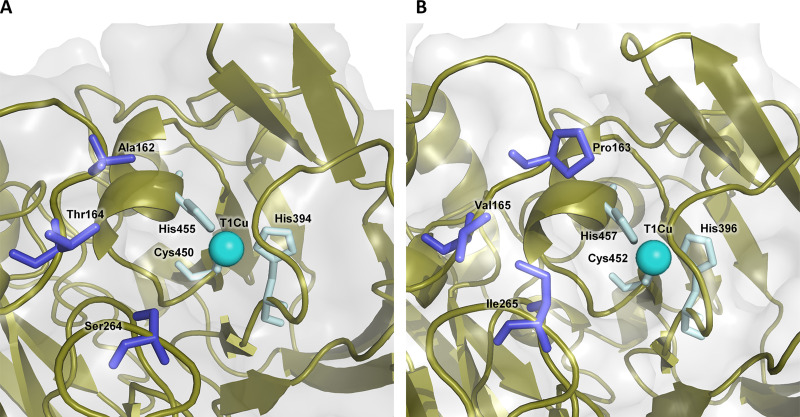
Details of the catalytic pocket in modern and ancestral laccases. (A) OB-1 modern evolved laccase. (B) LacAnc100 resurrected laccase. Protein surface is shown in gray and secondary motifs in olive cartoon. Residues submitted to saturation mutagenesis are represented as dark blue sticks, while residues involved in the first coordination sphere of the T1Cu are marked as light cyan sticks. T1Cu is represented as a cyan sphere. The model of LacAnc100 was made using the Phyre2 server (Protein Homology/analogY Recognition Engine V 2.0), available at www.sbg.bio.ic.ac.uk/phyre2. OB-1 mutations were modeled on PDB 5ANH by PyMOL (Schrodinger, LLC [http://www.pymol.org]).

To increase the activity of LacAnc100 toward 1,3-cyclopentanedione, we performed a small directed-evolution campaign (∼5,300 screened clones) that combined classical directed evolution (i.e., mutagenic PCR) with structure-guided evolution by combinatorial saturation mutagenesis and site-directed recombination. We first constructed a random point mutagenesis library on the whole LacAnc100 gene (excluding the signal peptide). With a mutational frequency of 1 to 4 mutations per kilobase (confirmed by sequencing of random variants) and after screening ∼1,600 clones, no improved variants were found, which suggests that the desired activity might possibly require remodeling of the active site and may not be accessible by point mutagenesis. Thus, we decided to turn to structure-guided evolution by mapping residues potentially involved in β-diketone binding at the T1Cu site of LacAnc100 (Fig. 7). Our model pointed to several residues at the second coordination sphere of the T1Cu site (Pro163, Val165, and Ile265) as potential candidates for combinatorial saturation mutagenesis (CSM) (29, 48, 49). We constructed two independent CSM libraries: library I (Pro163-Ile265) and library II (Val165-Ile265). After screening ∼3,200 clones, we identified two improved variants, P163R-I265I and V165R-I265F, from libraries I and II, respectively. To analyze potential synergies between mutations P163R, V165R, and I265F, an additional *in vivo* site-directed recombination (SDR) library was constructed and screened (480 clones, Fig. S4B), identifying the P163R-V165R mutant (Fig. S5). This double mutant produced oxidation rates for 1,3-cyclopentanedione that were 160% of those of the parental LacAnc100, representing a promising departure point for the future engineering of a competent laccase that acts on β-diketones.

**Conclusions.** In this study, we combined ancestral resurrection with the use of directed-evolution toolboxes to demonstrate that Mesozoic laccases can be generated and engineered in the laboratory. The improved expression, acid stability, and altered pH activity profiles of the ancestral laccase are thought to be a consequence of the adaptive advantages acquired during the Permian-Triassic extinction. However, from a practical point of view, these characteristics have long been pursued when laccases have been engineered for industrial use. Indeed, the combination of ancestral resurrection and directed evolution may allow a new generation of customized biocatalysts to be designed, and, by ultimately starting from virgin templates with no potentially disruptive mutations, the standard restraints in proteins engineered by directed evolution may be overcome. Additionally, the combination of the ancestral laccases described in this study with new mutations discovered by computational and directed evolution in modern counterparts could lead to the design of customized laccases with improved performances in terms of pH stability, activity, and expression (26, 27, 35).

## MATERIALS AND METHODS

### Strains and chemicals.

All chemicals were reagent-grade purity. The chemicals 2,2’-azino-bis(3-ethylbenzothiazoline-6-sulfonic acid (ABTS), 2.6-dimethoxyphenol (DMP), sinapic acid, guaiacol, potassium octacyanomolybdate(IV) (K_4_Mo[CN]_8_), and 1,3-cyclopentanedione and the yeast transformation kit were purchased from Sigma (St. Louis, MO, USA). Zymoprep Yeast Plasmid miniprep and Zymoclean gel DNA recovery kit were from Zymo Research (Orange, CA). A NucleoSpin Plasmid kit was purchased from Macherey-Nagel (Düren, Germany). The uracil-independent and ampicillin resistance shuttle vector pJRoC30 was obtained from the California Institute of Technology (Caltech, CA, USA). The protease-deficient S. cerevisiae strain BJ5465 (α *ura3-52 trp1 leu2*Δ*1 his3*Δ*200 pep4*::HIS3 *prb1*Δ*1.6R can1* GAL) was from LGC Promochem (Barcelona, Spain). The Escherichia coli strain XL2-Blue competent cells and *Pfu* DNA polymerase were obtained from Agilent Technologies (Santa Clara, CA, USA). Restriction endonucleases BamHI and XhoI were purchased from New England Biolabs (Ipswich, MA, USA). Oligonucleotide primers were acquired from Isogen Life Science (Barcelona, Spain).

### Culture media.

Culture media were prepared with the ingredients and recipes described elsewhere (27).

### Reconstruction and resurrection of ancestral nodes.

**(i) Alignment, phylogeny, and ancestral sequence reconstruction.** Fungal laccase homologs were retrieved from the April 2015 release of the complete genome database available at the National Center for Biotechnology Information (NCBI) (50). We deleted those sequences that did not include the four sequence regions identified by Kumar and coworkers (51). These regions correspond to laccase signatures that differentiate them from the broader class of multicopper oxidases. All sequences, including terms such as “hypothetical,” “predicted,” or “putative,” as well as those sequences from insects or ascomycetes, were discarded, resulting in a set of 120. The sequences were aligned using MUSCLE (available at https://www.ebi.ac.uk/Tools/msa/muscle/) and a distance matrix was then generated using one minus the sequence similarity as the parameter to assess the evolutionary distance between two sequences. The distribution for the calculated distances revealed two different groups, and we found that the major clade of Basidiomycota, the Agaricomycotina, class Agaricomycetes, was overrepresented relative to the rest. For phylogenetic analysis we employed the 87 sequences closest to the query since MrBayes efficiency strongly depends on sequence set size. Zygomycota and Ascomycota sequences were also included as outgroups to generate a rooted tree, making a total of 89 sequences (Table S1 in the supplemental material). The tree topology and the branch lengths of the trees were estimated from these sequences using the Bayesian method implemented in version MrBayes 3.1.2 (available at http://mrbayes.sourceforge.net/). This analysis used the JTT model and two independent Markov-chain Monte Carlo runs, each with four chains, and it was performed for 2,113,000 generations to ensure adequate convergence (0.04). The nodes obtained in the tree had posterior probabilities higher than 0.8, with very few exceptions. We targeted nodes 95, 98, and 100 in order to explore different alternatives of the tree, the three of them with probabilities close to unity (Fig. S1). The sequence reconstruction was performed using PAML version 4.4e (available at http://abacus.gene.ucl.ac.uk/software/paml.html) and with the WAG evolutionary model (available at http://www.ebi.ac.uk/goldman-srv/WAG/) (Fig. S2). Subsequently, the genes of the proteins encoded by the most probable sequences at each of the three nodes were synthesized to initiate the experiments for protein resurrection (i.e., functionally expressed in S. cerevisiae).

Accordingly, the DNAs encoding laccase ancestor node 95 (LacAnc95), laccase ancestor node 98 (LacAnc98), and laccase ancestor node 100 (LacAnc100) were synthesized by ATG:biosynthetics (Merzhausen, Germany).

**(ii) Fusion of ancestral laccase with different signal peptides and expression in S. cerevisiae.** LacAnc95, LacAnc98, and LacAnc100 were fused to different signal peptides for their functional expression and secretion in S. cerevisiae. The following signal sequences were tested: α factor prepro-leader from PM1L evolution, the α factor prepro-leader from Pycnoporus cinnabarinus laccase (PcL) evolution, and the chimeric preαproK from aryl alcohol oxidase (AAO) evolution (32, 36–38). To fuse LacAnc95, LacAnc98, and LacAnc100 to α^PM1^and α^PcL^, the laccase gene was amplified from a pUC derivative using sense oligonucleotides OB1_95F, OB1_98F, and OB1_100F for α^PM1^ and 3PO_95F, 3PO_98F, and 3PO_100F for α^PcL^ and antisense oligonucleotide RMLC for all cases (Table 3). The α-factor-OB1 (α^PM1^) (89 residues, including the STE13 cleavage site EAEA) was obtained from pJRoC30-OB-1 using the sense oligonucleotide RMLN primer and antisense oligonucleotides OB1_95R, OB1_98R, and OB1_100R. The α-factor-3PO (α^PcL^) (89 residues, including the STE13 cleavage site EAEA) was obtained from pJRoC30-3PO using sense oligonucleotide RMLN and antisense oligonucleotides 3PO_95R, 3PO_98R, and 3PO_100R (Table 3). The three node sequences were synthesized in a pUC derivative with the signal peptide preαproK-AAO. In order to insert constructions into the pJRoC30 vector, sense oligonucleotide RMLN and antisense oligonucleotide RMLC were used (Table 3). The amplification reactions were carried out in a Mycycler thermal cycler (Bio-Rad, USA). PCRs were performed in a final volume of 50 μl containing 250 nM each primer, 10 ng of template, dNTPs at 200 μM each, 3% (vol/vol) dimethyl sulfoxide (DMSO), and 0.02 U/μl of iProof high-fidelity polymerase. The PCR program for mature genes was 98°C for 30 s (1 cycle); 98°C for 10 s, 50°C for 25 s, and 72°C for 60 s (28 cycles); and 72°C for 10 min (1 cycle). The PCR program for signal peptide was 98°C for 30 s (1 cycle); 98°C for 10 s, 45°C for 30 s, and 72°C for 15 s (28 cycles); and 72°C for 10 min (1 cycle). PCR fragments were cleaned, concentrated, and loaded onto a low-melting-point preparative agarose gel (Bio-Rad, Hercules, CA), and then purified using the Zymoclean gel DNA recovery kit (Zymo Research, Orange, CA). PCR products were then mixed with the previously linearized vector pJRoC30 with XhoI and BamHI (at a PCR product/linearized plasmid ratio of 4:1) and transformed into competent S. cerevisiae cells (Yeast Transformation kit, Sigma). Reassembling *in vivo* upon yeast transformation was performed using ∼40-bp overhangs flanking each recombination area. Transformed cells were plated on SC drop-out plates and incubated for 3 days at 30°C. Colonies containing the whole autonomously replicating vector were grown, the laccase expression was induced, and supernatants were tested with laccase substrates (ABTS, DMP, sinapic acid, and guaiacol). Ethanol together with different culture temperatures were used to assess the expression levels of the different fusion genes (Fig. S3).

**TABLE 3:** Primers used for the fusion of signal peptides, library creation, and sequencing

Primer	Sequence 5′–3′^*a*^
RMLN	CCTCTATACTTTAACGTCAAGG
RMLC	GGGAGGGCGTGAATGTAAGC
OB1_95F	GAGAGACTGAAGCTGAATTCGCAGCGACTGTCGTTTATGA
OB1_95R	*TCATAAACGACAGTCGCTGC*GAATTCAGCTTCAGTCTCTC
OB1_98F	GAGAGACTGAAGCTGAATTCGCAGCAACCGTTGTGTATGA
OB1_98R	*TCATACACAACGGTTGCTGC*GAATTCAGCTTCAGTCTCTC
OB1_100F	GAGAGACTGAAGCTGAATTCGCGATAGGACCTGTGGCAGA
OB1_100R	*TCTGCCACAGGTCCTATCGC*GAATTCAGCTTCAGTCTCTC
3PO_95F	GAGGGGCTGAAGCTGAATTCGCAGCGACTGTCGTTTATGA
3PO_95R	*TCATAAACGACAGTCGCTGC*GAATTCAGCTTCAGCCCCTC
3PO_98F	GAGGGGCTGAAGCTGAATTCGCAGCAACCGTTGTGTATGA
3PO_98R	*TCATACACAACGGTTGCTGC*GAATTCAGCTTCAGCCCCTC
3PO_100F	*GAGGGGCTGAAGCTGAATTC*GCGATAGGACCTGTGGCAGA
3PO_100R	*TCTGCCACAGGTCCTATCGC*GAATTCAGCTTCAGCCCCTC
163A-F	GCTCCCCAGTTGGCCGCT**NDT**CCGCCCGTCCCAGATAGCAC
163B-F	GCTCCCCAGTTGGCCGCT**VGH**CCGCCCGTCCCAGATAGCAC
163C-F	GCTCCCCAGTTGGCCGCT**TGG**CCGCCCGTCCCAGATAGCAC
163A-R	GTGCTATCTGGGACGGGCGG**AHN**AGCGGCCAACTGGGGAGC
163B-R	GTGCTATCTGGGACGGGCGG**DCB**AGCGGCCAACTGGGGAGC
163C-R	GTGCTATCTGGGACGGGCGG**CCA**AGCGGCCAACTGGGGAGC
265A-F	GATAAGAGCGAATCCAAAT**NDT**GGAACGACAGGATTCGCA
265B-F	GATAAGAGCGAATCCAAAT**VGH**GGAACGACAGGATTCGCA
265C-F	GATAAGAGCGAATCCAAAT**TGG**GGAACGACAGGATTCGCA
265A-R	TGCGAATCCTGTCGTTCC**AHN**ATTTGGATTCGCTCTTATC
265B-R	TGCGAATCCTGTCGTTCC**DCB**ATTTGGATTCGCTCTTATC
265C-R	TGCGAATCCTGTCGTTCC**CCA**ATTTGGATTCGCTCTTATC
165A-F	CCAGTTGGCCGCTGCGCCGCCC**NDT**CCAGATAGCACCCTA
165B-F	CCAGTTGGCCGCTGCGCCGCCC**VGH**CCAGATAGCACCCTA
165C-F	CCAGTTGGCCGCTGCGCCGCCC**TGG**CCAGATAGCACCCTA
165A-R	TAGGGTGCTATCTGG**AHN**GGGCGGCGCAGCGGCCAACTGG
165B-R	TAGGGTGCTATCTGG**DCB**GGGCGGCGCAGCGGCCAACTGG
165C-R	TAGGGTGCTATC**TGG**CCAGGGCGGCGCAGCGGCCAACTGG
163165wtRev	GTGCTATCTGG**GAC**GGGCGG**CGC**AGCGGCCAACTGGGGAGC
163165mutRev	GTGCTATCTGG**ACG**GGGCGG**ACG**AGCGGCCAACTGGGGAGC
163165wtFor	GCTCCCCAGTTGGCCGCT**GCG**CCGCCC**GTC**CCAGATAGCAC
163165mutFor	GCTCCCCAGTTGGCCGCT**CGT**CCGCCC**CGT**CCAGATAGCAC
265Rev	TGCGAATCCTGTCGTTCC**RAWA**TTTGGATTCGCTCTTATC
265For	GATAAGAGCGAATCCAAAT**WTY**GGAACGACAGGATTCGCA
FS	ACGACTTCCAGGTCCCTGACCAAGC
RS	TCAATGTCCGCGTTCGCAGGGA
Lac100Forward	CGTTAGCCGTGATAAACGTAGAGCAGGGAAA
Lac100Foward2	GATTCTTTCAACACGGGACAAATTGGGCTGACG
Lac100Mut	GCGATAGGACCTGTGGCAGACCTTCACATC
Lac100HF	GATGTGAAGGTCTGCCACAGGTCCTATCGC

a Overhangs for pJRoC30, for cloning, are underlined. Overhangs for laccase genes, for cloning, are shown in italics and underlined. Codons including targeted positions for saturation or recombination are shown in bold. Mixed bases are represented under the following code: W = A/T; Y = C/T; K = G/T; R = A/G.

### Biochemical characterization.

**(i) Laccase production and purification.** Laccase production and purification methods were adapted from previous protocols with minor modifications (32, 40). The purified laccases were analyzed by SDS-polyacrylamide gel electrophoresis (SDS-PAGE) in their glycosylated and deglycosylated forms. The purified enzymes were deglycosylated using peptide:*N*-glycanase (PNGase F) (New England Biolabs, Ipswich, MA, USA). All protein concentrations were determined using the Bio-Rad protein reagent and bovine serum albumin as a standard.

**(ii) pH activity profile.** Appropriate dilutions of supernatants were prepared with the help of a robot in such a way that aliquots of 20 μl give rise to a linear response in kinetic mode. Vessels containing 100 mM Britton and Robinson buffer with 1 mM ABTS, 5 mM DMP, 5 mM K_4_Mo(CN)_8_, and 50 mM guaiacol were prepared at pH values of 2.0, 3.0, 4.0, 5.0, 6.0, 7.0, 8.0, and 9.0. The assay started when the different reactions mixtures of ABTS, DMP, K_4_Mo(CN)_8_, and guaiacol were added to each well of a 96-well plate. The activities were measured at room temperature in triplicate in kinetic mode, and the relative activity (expressed in %) is based on the maximum activity for each variant in the assay.

**(iii) pH stability profile.** Appropriate dilutions of supernatants were prepared with the help of a robot in such a way that aliquots of 20 μl give rise to a linear response in kinetic mode. Samples of 500 μl were incubated at different times over a range of pH values in 100 mM Britton and Robinson buffer (2.0, 3.0, 4.0, 5.0, 6.0, 7.0, 8.0, and 9.0). Samples of 20 μl were removed at different times (0, 4, 24, 48, 72, and 144 h) and subjected to the same ABTS-based colorimetric assay described above for the screening. The activities were measured in triplicate at room temperature in kinetic mode and laccase activity was normalized to the activity value at time zero for the highest activity obtained.

**(iv) Kinetic thermostability and half-life (*t*_1/2_).** Appropriate dilutions of supernatants were prepared at pH 3.0 and 6.0 with the help of a robot in such a way that aliquots of 20 μl give rise to a linear response in kinetic mode. Aliquots of 50 μl (from both selected mutants and parent types) were used for each point of the incubation. A fixed temperature of 70°C and 50°C was established for pH 6.0 and 3.0, respectively. Samples were removed at different times (0, 5, 15, 25, 35, 55, 75, 95, and 120 min) from the thermocycler (Mycycler; Bio-Rad) and chilled on ice for 10 min. After this, samples of 20 μl were removed and incubated at room temperature for 5 min. Finally, samples were subjected to the same ABTS-based colorimetric assay described above for the screening. Thermostability values were deduced from the ratio between the residual activities incubated at different temperature points and the initial activity at room temperature.

**(v) Steady-state kinetic constants.** ABTS, DMP, sinapic acid, and guaiacol kinetic constants for pure laccases were estimated in 100 mM sodium phosphate/citrate buffer (at pH 4.0 for ABTS and at pH 5.0 for DMP, sinapic acid, and guaiacol). Different concentrations for ABTS (0.0025, 0.005, 0.0075, 0.01, 0.0125, 0.025, 0.05, 0.075, and 0.1 mM), DMP (0.005, 0.01, 0.025, 0.05, 0.1, 0.25, 0.5, and 1 mM), sinapic acid (0.005, 0.01, 0.025, 0.05, 0.1, 0.25, 0.5, 0.75, and 1 mM), and guaiacol (0.1, 0.25, 0.5, 1, 3, 5, 7, 10, and 30 mM) were mixed with appropriate dilutions of pure OB-1 (0.30 nM for ABTS, 0.60 nM for DMP, 6 nM for sinapic acid, and 6 nM for guaiacol), LacAnc98 (1.61 nM for ABTS, 8.04 nM for DMP, 32.17 nM for sinapic acid, and 16.08 nM for guaiacol), and LacAnc100 (0.7 nM for ABTS, 1.4 nM for DMP, 13.8 nM for sinapic acid, and 10 nM for guaiacol). Reactions were performed at room temperature in triplicate, and substrate oxidations were followed through spectrophotometric changes (Ɛ_418_ ABTS^+•^ = 36,000 M^−1 ^cm^−1^; Ɛ_469_ DMP = 27,500 M^−1 ^cm^−1^; Ɛ_512_ sinapic acid = 14,066 M^−1 ^cm^−1^; and Ɛ_470_ guaiacol = 12,100 M^−1 ^cm^−1^). To calculate the *K*_m_ and *k*_cat_ values, the average *V*_max_ was represented against substrate concentration and fitted to a single rectangular hyperbola function with SigmaPlot 10.0, where parameter a was equal to *k*_cat_ and parameter b was equal to *K*_m_.

### Directed evolution.

**(i) Random point mutagenesis library.** A random mutagenic PCR (excluding the signal peptide) of LacAnc100 was designed by MORPHING (52). The reaction mixture was prepared in a final volume of 50 μl containing 10 ng of template, 90 nM of each primer (Lac100Mut and RMLC, Table 3), 0.3 mM dNTPs (0.075 mM each), 3% (vol/vol) dimethyl sulfoxide (DMSO), 0.03 mM MnCl_2_, 1.5 mM MgCl_2_, and 0.05 U/μl *Taq* DNA polymerase. The PCR program was as follows: 1 cycle of 94°C for 2 min; 28 cycles of 94°C for 45 s, 50°C for 25 s, and 72°C for 90 s; and 1 cycle of 72°C for 10 min. The signal peptide α factor-PcL (LacAnc100 signal peptide) was amplified by high-fidelity PCR in a final volume of 50 μl containing 250 nM each primer (RMLN and Lac100HF) [Table 3], 10 ng of template, dNTPs at 200 μM each, 3% (vol/vol) dimethyl sulfoxide (DMSO), and 0.02 U/μl of iProof high-fidelity polymerase. The PCR program was as follows: 1 cycle of 98°C for 30 s; 28 cycles of 98°C for 10 s, 45°C for 30 s, and 72°C for 15 s; and 1 cycle of 72°C for 10 min. PCR products were treated as reported above and then mixed with the previously linearized vector pJRoC30 with XhoI and BamHI (at a PCR product/linearized plasmid ratio of 4:1) and transformed into S. cerevisiae competent cells (Sigma yeast transformation kit). Reassembling *in vivo* upon yeast transformation was performed using ∼40-bp overhangs flanking each recombination area. Transformed cells were plated on SC drop-out plates and incubated for 3 days at 30°C. Colonies containing the whole autonomously replicating vector were subjected to high-throughput screening as described below. Twenty random clones from the library were selected, plasmids were extracted, and sequencing was performed to verify the mutational loading.

**(ii) Combinatorial saturation mutagenesis.** Two independent CSM libraries were constructed: library I (Pro163-Ile265) and library II (Val165-Ile265). CSM libraries were constructed by the 22c-trick, combining three primers that allowed a degeneracy of 22 unique codons for the 20 natural amino acids to occur, decreasing the number of variants under study (53). In library I, position 163 was saturated using oligonucleotides 163A-F, 163B-F, and 163C-F (sense) and 163A-R, 163B-R, and 163C-R (antisense), whereas for position 265, oligonucleotides 265A-F, 265B-F, and 265C-F (sense) and 265A-R, 265B-R, and 265C-R (antisense) were used (Table 3). In library II, position 165 was saturated using oligonucleotides 165A-F, 165B-F, and 165C-F (sense) and 165A-R, 165B-R, and 165C-R (antisense), whereas for position 265, we used the same oligonucleotides reported in library I (Table 3). PCRs were performed in a final volume of 50 μl containing 250 nM each primer, 10 ng of template, dNTPs at 200 μM each, 3% (vol/vol) dimethyl sulfoxide (DMSO), and 0.02 U/μl of iProof high-fidelity polymerase. The PCR program was 1 cycle of 98°C for 30 s; 28 cycles of 98°C for 10 s, 50°C for 25 s, and 72°C for 60 s; and 1 cycle of 72°C for 10 min. Aliquots of 200 ng of each PCR product (400 in total) were transformed with linearized plasmid (100 ng) into S. cerevisiae competent cells. PCR products from each library were transformed independently. PCR products were treated as reported above and then mixed in equal portions with the previously linearized vector pJRoC30 with XhoI and BamHI (at a PCR product/linearized plasmid ratio of 4:1) and transformed into S. cerevisiae competent cells (Yeast Transformation kit, Sigma). Reassembling *in vivo* upon yeast transformation was performed using ∼40-bp overhangs flanking each recombination area. Transformed cells were plated on SC drop-out plates and incubated for 3 days at 30°C. Colonies containing the whole autonomously replicating vector were subjected to high-throughput screening as described below.

**(iii) *In vivo* site-directed recombination library.** The SDR method applied in reference 54 was adapted with several modifications. LacAnc100 was used as the DNA template to insert all possible mutations/reversions, P163R, V165R, and I265F, by SDR. PCR1 used oligonucleotide sense RMLN and an equimolar mix of antisense oligonucleotides 163165wtRev and 163165mutRev (Table 3). PCR2 was performed with an equimolar mix of oligonucleotides sense 163165wtFor and 163165mutFor and oligonucleotide antisense 265Rev. PCR3 used sense oligonucleotide 265For and antisense oligonucleotide RMLC (Table 3, Fig. S4B). PCRs were performed in a final volume of 50 μl containing 250 nM of each primer, 10 ng of template, dNTPs at 200 μM each, 3% (vol/vol) dimethyl sulfoxide (DMSO), and 0.02 U/μl of iProof high-fidelity polymerase. The PCR program was as follows: 1 cycle of 98°C for 30 s; 28 cycles of 98°C for 10 s, 50°C for 25 s, and 72°C for 60 s; and 1 cycle of 72°C for 10 min. PCR products were treated as reported above and then mixed in equal amounts, 200 ng from each of the three PCRs products, and transformed with linearized plasmid (150 ng) into competent cells (Sigma yeast transformation kit). Reassembling and recombination *in vivo* upon yeast transformation was performed using ∼40-bp overhangs flanking each recombination area. Transformed cells were plated on SC drop-out plates and incubated for 3 days at 30°C. Colonies containing the whole autonomously replicating vector were subjected to high-throughput screening as described below.

**(iv) High-throughput screening.** Individual clones were picked and inoculated in sterile 96-well plates (Greiner Bio-One, GmbH, Germany), referred to as master plates, containing 200 μl of selective expression medium (SEM) per well. The composition of SEM is described elsewhere (27). In addition to the regular SEM, the SEM without ethanol supply was also used to test the expression of the different fusion genes and signal peptides (Fig. S3). In each plate, column number 6 was inoculated with the parental type, and one well (H1-control) was inoculated with S. cerevisiae transformed with plasmid pJRoC30-AAO (aryl-alcohol oxidase without activity versus 2,2’-azino-bis[3-ethylbenzothiazoline-6-sulfonic acid] [ABTS] or 1,3-cyclopentanedione). Plates were sealed with Parafilm to prevent evaporation and incubated at 30°C, 220 rpm, and 80% relative humidity in a humidity shaker (Minitron, Infors, Switzerland) for 3 days. The master plates were centrifuged (5810R centrifuge; Eppendorf, Hamburg, Germany) for 10 min at 2,500 × *g* and 4°C. Aliquots of the supernatants were transferred from the master plates to two replica plates with the aid of a robotic station Freedom EVO liquid handler (Tecan, Switzerland), 80 μl of mixture for β-diketone screening and 20 μl for the ABTS screening. To estimate the β-diketone activity, 120 μl of 100 mM citrate phosphate buffer (pH 5.0) containing 50 mM 1,3-cyclopentanedione was added to the replica plates containing 80 μl of supernatant. Plates were stirred briefly and the absorption at 450 nm was measured in endpoint mode in the plate reader (SPECTRAMax Plus 384, Molecular Devices, Sunnyvale, CA). Afterward the plates were sealed with Parafilm and incubated in a shaker at 35°C. Incubation took place for 40 h and then plates were measured again. The activities were calculated from the difference between the absorption after incubation and that of the initial measurement normalized against the parental type, LacAnc100, in the corresponding plate. To estimate the ABTS activity, 180 μl of 100 mM citrate phosphate buffer (pH 4.0) containing 1 mM ABTS was added to the replica plates containing 20 μl of supernatant. Plates were stirred briefly and the absorption at 418 nm (ε_418_ ABTS^•+^ = 36,000 M^−1 ^cm^−1^) was followed in kinetic mode in the plate reader. To rule out false positives, two consecutive rescreenings were carried out according to the protocol previously reported (32). A total number of 5,280 clones were screened: 1,600 clones from the mutagenic PCR library, 1,600 clones from CSM library I, 1,600 clones from CSM library II, and 480 clones from the SDR library.

### DNA sequencing.

Plasmids containing laccase variant genes were sequenced by GATC-Biotech. The samples, prepared with 5 μl of 100 ng/μl plasmid and 5 μl of each 5 μM primer, were as follows: RMLN, FS, RS, and RMLC for extant laccase and RMLN, LacAnc100Forward, LacAnc100Forward2, and RMLC for ancestral laccase and mutants (Table 3).

### Protein modeling.

The structural model of wtPM1L at a resolution of 2.49 Å (1 Å = 0.1 nm) (PDB code 5ANH) was used to map the mutations. LacAnc100 was modeled by the Phyre2 (Protein Homology/analogY Recognition Engine V 2.0) server, available at www.sbg.bio.ic.ac.uk/phyre2.

## Supplementary Material

Supplemental file 1

## References

[1] Pauling L, Zuckerkandl E, Henriksen T, Lövstad R 1963 Molecular “restoration studies” of extinct forms of life. Acta Chem Scand 17(Suppl):9–16. doi:10.3891/acta.chem.scand.17s-0009.

[2] Malcolm BA, Wilson KP, Matthews BW, Kirsch JF, Wilson AC 1990 Ancestral lysozymes reconstructed, neutrality tested, and thermostability linked to hydrocarbon packing. Nature 345:86–89. doi:10.1038/345086a0. 2330057

[3] Stackhouse J, Presnell SR, McGeehan GM, Nambiar KP, Benner SA 1990 The ribonuclease from an extinct bovid ruminant. FEBS Lett 262:104–106. doi:10.1016/0014-5793(90)80164-e. 2318301

[4] Jermann TM, Opitz JG, Stackhouse J, Benner SA 1995 Reconstructing the evolutionary history of the artiodactyl ribonuclease superfamily. Nature 374:57–59. doi:10.1038/374057a0. 7532788

[5] Ayuso-Fernández I, Martínez AT, Ruiz-Dueñas FJ 2017 Experimental recreation of the evolution of lignin-degrading enzymes from the Jurassic to date. Biotechnol Biofuels 10:67. doi:10.1186/s13068-017-0744-x. 28331543 PMC5356311

[6] Gomez-Fernandez BJ, Garcia-Ruiz E, Martin-Diaz J, Gomez de Santos P, Santos-Moriano P, Plou FJ, Ballesteros A, Garcia M, Rodriguez M, Risso VA, Sanchez-Ruiz JM, Whitney SM, Alcalde M 2018 Directed—in vitro—evolution of Precambrian and extant Rubiscos. Sci Rep 8:5532. doi:10.1038/s41598-018-23869-3. 29615759 PMC5883036

[7] Risso VA, Sanchez-Ruiz JM, Ozkan SB 2018 Biotechnological and protein-engineering implications of ancestral protein resurrection. Curr Opin Struct Biol 51:106–115. doi:10.1016/j.sbi.2018.02.007. 29660672

[8] Babkova P, Sebestova E, Brezovsky J, Chaloupkova R, Damborsky J 2017 Ancestral haloalkane dehalogenases show robustness and unique substrate specificity. Chembiochem 18:1448–1456. doi:10.1002/cbic.201700197. 28419658

[9] Zakas PM, Brown HC, Knight K, Meeks SL, Spencer HT, Gaucher EA, Doering CB 2017 Enhancing the pharmaceutical properties of protein drugs by ancestral sequence reconstruction. Nat Biotechnol 35:35–37. doi:10.1038/nbt.3677. 27669166 PMC5225049

[10] Whitfield JH, Zhang WH, Herde MK, Clifton BE, Radziejewski J, Janovjak H, Henneberger C, Jackson CJ 2015 Construction of a robust and sensitive arginine biosensor through ancestral protein reconstruction. Protein Sci 24:1412–1422. doi:10.1002/pro.2721. 26061224 PMC4570536

[11] Cox VE, Gaucher EA 2014 Engineering proteins by reconstructing evolutionary adaptive paths, p 353–363. *In* Gillam EMJ, Copp JN, Ackerley D (ed), Directed evolution library creation: methods and protocols. Springer New York, New York, NY. 10.1007/978-1-4939-1053-3_24 25055790

[12] Risso VA, Gavira JA, Mejia-Carmona DF, Gaucher EA, Sanchez-Ruiz JM 2013 Hyperstability and substrate promiscuity in laboratory resurrections of Precambrian β-lactamases. J Am Chem Soc 135:2899–2902. doi:10.1021/ja311630a. 23394108

[13] Akanuma S, Nakajima Y, Yokobori SI, Kimura M, Nemoto N, Mase T, Miyazono KI, Tanokura M, Yamagishi A 2013 Experimental evidence for the thermophilicity of ancestral life. Proc Natl Acad Sci U S A 110:11067–11072. doi:10.1073/pnas.1308215110. 23776221 PMC3703998

[14] Risso VA, Gavira JA, Sanchez-Ruiz JM 2014 Thermostable and promiscuous Precambrian proteins. Environ Microbiol 16:1485–1489. doi:10.1111/1462-2920.12319. 25009840

[15] Gaucher EA, Govindarajan S, Ganesh OK 2008 Palaeotemperature trend for Precambrian life inferred from resurrected proteins. Nature 451:704–707. doi:10.1038/nature06510. 18256669

[16] Alcalde M 2017 When directed evolution met ancestral enzyme resurrection. Microb Biotechnol 10:22–24. doi:10.1111/1751-7915.12452. 27863072 PMC5270717

[17] Risso VA, Sanchez-Ruiz JM 2017 Resurrected ancestral proteins as scaffolds for protein engineering, p 229–255. *In* Alcalde M (ed), Directed enzyme evolution: advances and applications, Springer International Publishing, Cham, Switzerland.

[18] Alcalde M 2015 Engineering the ligninolytic enzyme consortium. Trends Biotechnol 33:155–162. doi:10.1016/j.tibtech.2014.12.007. 25600621

[19] Arnaud CH 2013 Bringing ancient proteins to life. Chem Eng News 91:38–39.

[20] Cañas AI, Camarero S 2010 Laccases and their natural mediators: biotechnological tools for sustainable eco-friendly processes. Biotechnol Adv 28:694–705. doi:10.1016/j.biotechadv.2010.05.002. 20471466

[21] Alcalde M 2007 Laccases: biological functions, molecular structure and industrial applications, p 461–476. *In* Polaina J, MacCabe AP (ed), Industrial enzymes. Structure, function and applications, Springer Netherlands, Dordrecht, Netherlands.

[22] Morozova OV, Shumakovich GP, Shleev SV, Iaropolov AI 2007 Laccase-mediator systems and their applications: a review. Appl Biochem Microb 43:583–597. doi:10.1134/S0003683807050055. 18038679

[23] Mate DM, Alcalde M 2017 Laccase: a multi-purpose biocatalyst at the forefront of biotechnology. Microb Biotechnol 10:1457–1467. doi:10.1111/1751-7915.12422. 27696775 PMC5658592

[24] Xu F 2005 Applications of oxidoreductases: recent progress. Ind Biotechnol 1:38–50. doi:10.1089/ind.2005.1.38.

[25] Solomon EI, Sundaram UM, Machonkin TE 1996 Multicopper oxidases and oxygenases. Chem Rev 96:2563–2606. doi:10.1021/cr950046o. 11848837

[26] Mateljak I, Monza E, Lucas MF, Guallar V, Aleksejeva O, Ludwig R, Leech D, Shleev S, Alcalde M 2019 Increasing redox potential, redox mediator activity, and stability in a fungal laccase by computer-guided mutagenesis and directed evolution. ACS Catal 9:4561–4572. doi:10.1021/acscatal.9b00531.

[27] Mateljak I, Rice A, Yang K, Tron T, Alcalde M 2019 The generation of thermostable fungal laccase chimeras by SCHEMA-RASPP structure-guided recombination in vivo. ACS Synth Biol 8:833–843. doi:10.1021/acssynbio.8b00509. 30897903

[28] Pardo I, Rodríguez-Escribano D, Aza P, de Salas F, Martínez AT, Camarero S 2018 A highly stable laccase obtained by swapping the second cupredoxin domain. Sci Rep 8:15669. doi:10.1038/s41598-018-34008-3. 30353103 PMC6199291

[29] Pardo I, Santiago G, Gentili P, Lucas F, Monza E, Medrano FJ, Galli C, Martinez AT, Guallar V, Camarero S 2016 Re-designing the substrate binding pocket of laccase for enhanced oxidation of sinapic acid. Catal Sci Technol 6:3900–3910. doi:10.1039/C5CY01725D.

[30] de Salas F, Aza P, Gilabert JF, Santiago G, Kilic S, Sener ME, Vind J, Guallar V, Martínez AT, Camarero S 2019 Engineering of a fungal laccase to develop a robust, versatile and highly-expressed biocatalyst for sustainable chemistry. Green Chem 21:5374–5385. doi:10.1039/C9GC02475A.

[31] Mate DM, Alcalde M 2015 Laccase engineering: from rational design to directed evolution. Biotechnol Adv 33:25–40. doi:10.1016/j.biotechadv.2014.12.007. 25545886

[32] Mate DM, Garcia-Burgos C, Garcia-Ruiz E, Ballesteros AO, Camarero S, Alcalde M 2010 Laboratory evolution of high-redox potential laccases. Chem Biol 17:1030–1041. doi:10.1016/j.chembiol.2010.07.010. 20851352

[33] Hedges SB, Kumar S (ed). 2009 The timetree of life. Oxford University Press, Oxford, United Kingdom.

[34] Nowak H, Schneebeli-Hermann E, Kustatscher E 2019 No mass extinction for land plants at the Permian–Triassic transition. Nat Commun 10:384–391. doi:10.1038/s41467-018-07945-w. 30674875 PMC6344494

[35] Gomez-Fernandez BJ, Risso VA, Sanchez-Ruiz JM, Alcalde M 2020 Consensus design of an evolved high-redox potential laccase. Front Bioeng Biotechnol 6 May. doi:10.3389/fbioe.2020.00354. PMC7218104 32435637

[36] Mateljak I, Tron T, Alcalde M 2017 Evolved α-factor prepro-leaders for directed laccase evolution in *Saccharomyces cerevisiae*. Microb Biotechnol 10:1830–1836. doi:10.1111/1751-7915.12838. 28805314 PMC5658585

[37] Viña-Gonzalez J, Gonzalez-Perez D, Ferreira P, Martinez AT, Alcalde M 2015 Focused directed evolution of aryl-alcohol oxidase in *Saccharomyces cerevisiae* by using chimeric signal peptides. Appl Environ Microbiol 81:6451–6462. doi:10.1128/AEM.01966-15. 26162870 PMC4542223

[38] Camarero S, Pardo I, Cañas AI, Molina P, Record E, Martinez AT, Martinez MJ, Alcalde M 2012 Engineering platforms for directed evolution of Laccase from *Pycnoporus cinnabarinus*. Appl Environ Microbiol 78:1370–1384. doi:10.1128/AEM.07530-11. 22210206 PMC3294479

[39] Bulter T, Alcalde M, Sieber V, Meinhold P, Schlachtbauer C, Arnold FH 2003 Functional expression of a fungal laccase in *Saccharomyces cerevisiae* by directed evolution. Appl Environ Microbiol 69:987–995. doi:10.1128/aem.69.2.987-995.2003. 12571021 PMC143632

[40] Mate DM, Garcia-Ruiz E, Camarero S, Shubin VV, Falk M, Shleev S, Ballesteros AO, Alcalde M 2013 Switching from blue to yellow: altering the spectral properties of a high redox potential laccase by directed evolution. Biocatal Biotransfor 31:8–21. doi:10.3109/10242422.2012.749463.

[41] Hollmann F, Arends I 2012 Enzyme initiated radical polymerizations. Polymers 4:759–793. doi:10.3390/polym4010759.

[42] Hollmann F, Gumulya Y, TöLle C, Liese A, Thum O 2008 Evaluation of the laccase from *Myceliophthora thermophila* as industrial biocatalyst for polymerization reactions. Macromolecules 41:8520–8524. doi:10.1021/ma801763t.

[43] Durand A, Lalot T, Brigodiot M, Maréchal E 2000 Enzyme-mediated initiation of acrylamide polymerization: reaction mechanism. Polymers 41:8183–8192. doi:10.1016/S0032-3861(00)00204-4.

[44] Singh A, Ma D, Kaplan DL 2000 Enzyme-mediated free radical polymerization of styrene. Biomacromolecules 1:592–596. doi:10.1021/bm005537j. 11710186

[45] Teixeira D, Lalot T, Brigodiot M, Maréchal E 1999 β-Diketones as key compounds in free-radical polymerization by enzyme-mediated initiation. Macromolecules 32:70–72. doi:10.1021/ma980872+.

[46] Lizotte JR, Long TE 2003 Stable free-radical polymerization of styrene in combination with 2-vinylnaphthalene initiation. Macromol Chem Phys 204:570–576. doi:10.1002/macp.200390020.

[47] Durand A, Lalot T, Brigodiot M, Maréchal E 2001 Enzyme-mediated radical initiation of acrylamide polymerization: main characteristics of molecular weight control. Polymer 42:5515–5521. doi:10.1016/S0032-3861(01)00041-6.

[48] Galli C, Gentili P, Jolivalt C, Madzak C, Vadalà R 2011 How is the reactivity of laccase affected by single-point mutations? Engineering laccase for improved activity towards sterically demanding substrates. Appl Microbiol Biotechnol 91:123–131. doi:10.1007/s00253-011-3240-4. 21468703

[49] Mohamad SB, Ong AL, Ripen AM 2008 Evolutionary trace analysis at the ligand binding site of laccase. Bioinformation 2:369–372. doi:10.6026/97320630002369. 18795108 PMC2533054

[50] NCBI Resource Coordinators. 2018 Database resources of the National Center for Biotechnology Information. Nucleic Acids Res 46:8–13. doi:10.1093/nar/gkv1290. PMC5753372 29140470

[51] Kumar SVS, Phale PS, Durani S, Wangikar PP 2003 Combined sequence and structure analysis of the fungal laccase family. Biotechnol Bioeng 83:386–394. doi:10.1002/bit.10681. 12800133

[52] Gonzalez-Perez D, Molina-Espeja P, Garcia-Ruiz E, Alcalde M 2014 Mutagenic organized recombination process by homologous *in vivo* grouping (MORPHING) for directed enzyme evolution. PLoS One 9:e90919. doi:10.1371/journal.pone.0090919. 24614282 PMC3948698

[53] Kille S, Acevedo-Rocha CG, Parra LP, Zhang ZG, Opperman DJ, Reetz MT, Acevedo JP 2013 Reducing codon redundancy and screening effort of combinatorial protein libraries created by saturation mutagenesis. ACS Synth Biol 2:83–92. doi:10.1021/sb300037w. 23656371

[54] Viña-Gonzalez J, Jimenez-Lalana D, Sancho F, Serrano A, Martinez AT, Guallar V, Alcalde M 2019 Structure-guided evolution of aryl alcohol oxidase from *Pleurotus eryngii* for the selective oxidation of secondary benzyl alcohols. Adv Synth Catal 361:2514–2525. doi:10.1002/adsc.201900134.

