# Supplementary material for: Ancestral Resurrection and Directed Evolution of Fungal Mesozoic Laccases
Source: Appl Environ Microbiol. 2020 Jul 2;86(14):e00778-20. doi: 10.1128/AEM.00778-20 (PMC7357490; doi:10.1128/AEM.00778-20)
Supplement: Supplemental file 1 [file AEM.00778-20-s0001.pdf]

Supplementary material for

Ancestral Resurrection and Directed Evolution of Fungal Mesozoic Laccases

Bernardo J. Gomez-Fernandez[1], Valeria A. Risso[2], Andres Rueda[3], Jose M. Sanchez-Ruiz[2] and Miguel Alcalde[1]*

[1]Department of Biocatalysis, Institute of Catalysis and Petrochemistry, CSIC, C/ Marie Curie 2, 28049, Madrid, Spain.

[2]Departamento de Química Física, Facultad de Ciencias, Universidad de Granada, 18071, Granada, Spain.

[3]INRS–Institut Armand-Frappier. Université du Québec. Laval. QC, Canada.

*Corresponding author: malcalde@icp.csic.es

Supplemental material contains 5 supplementary Figures and 1 supplementary Table.

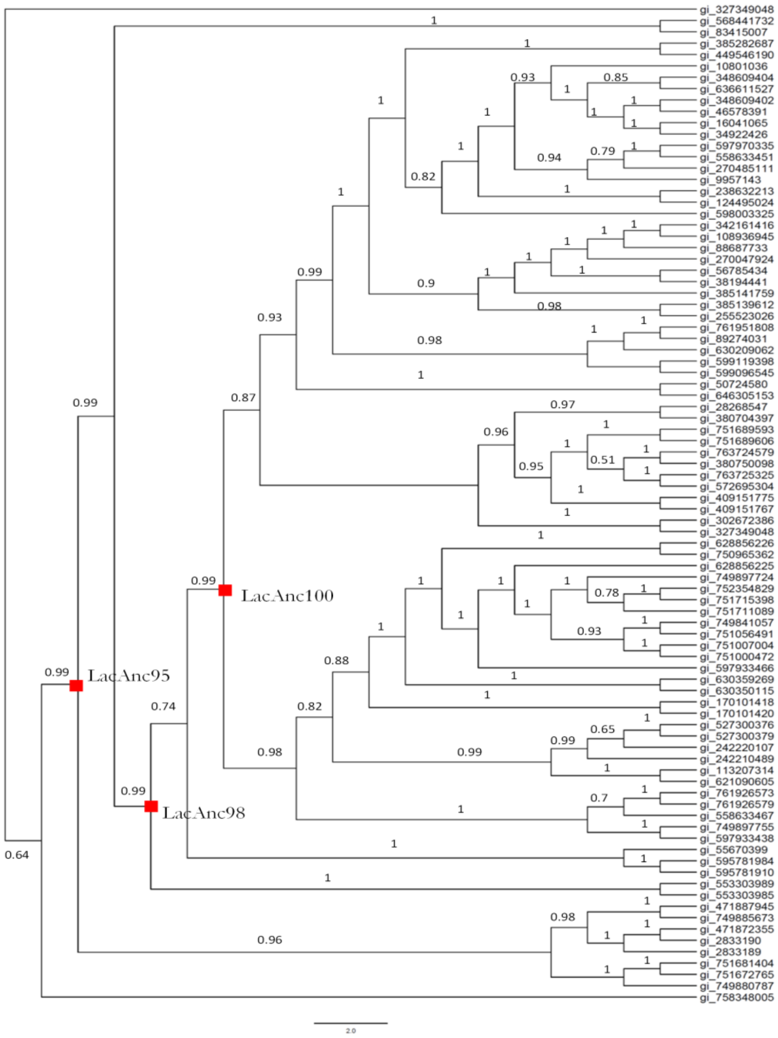

Figure S1. Inferenced evolutionary trees with the posterior probability at each node.

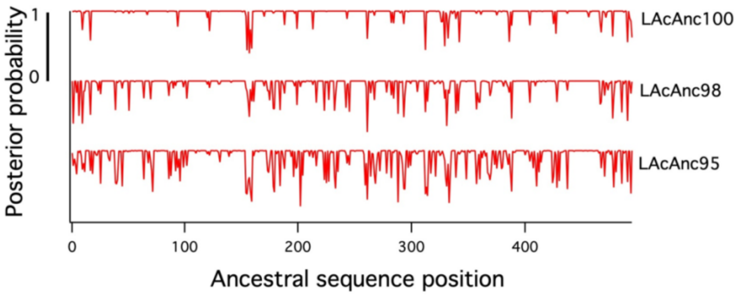

Figure S2. Robustness of the reconstructed ancestors as measure of the distribution of posterior probability.

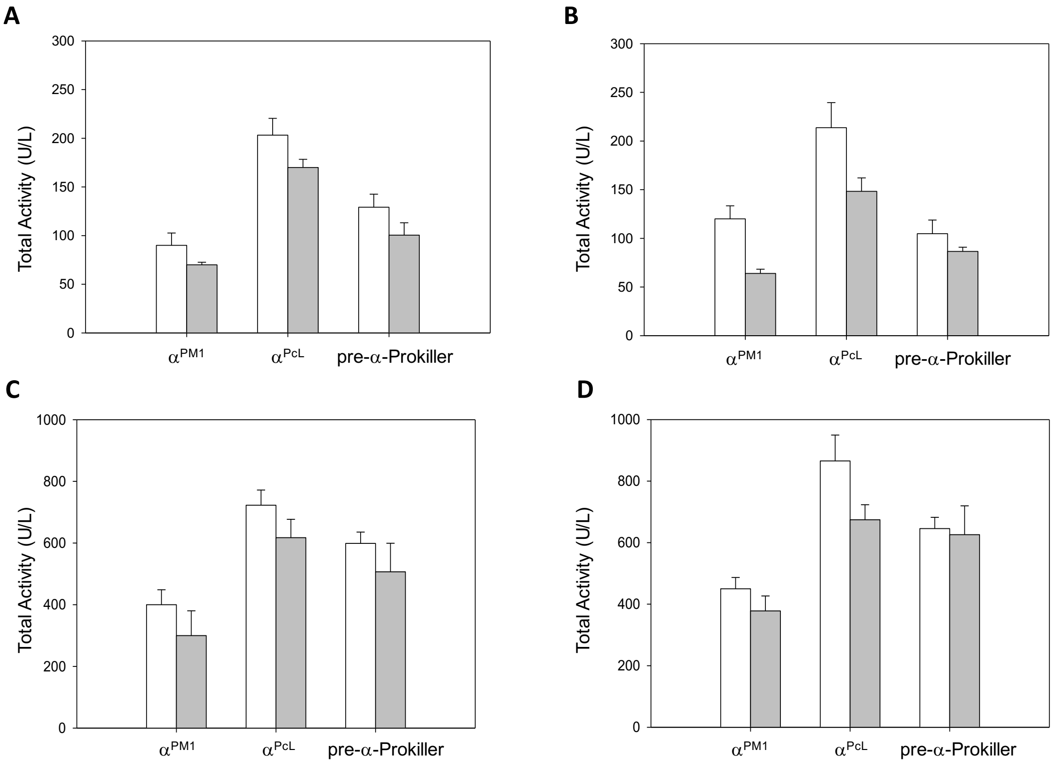

Figure S3. Ancestral laccase secretion with different signal peptides and conditions. A) LacAnc98 at 25°C; **B**) LacAnc98 at 30°; **C)** LacAnc100 at 25°C; **D)** LacAnc100 at 30°C. The x-axis represents the three different signal peptides assayed α^{PM1}, α^{PcL}, pre-α-prokiller. White bars, total activity in SEM media with ethanol; grey bars, total activity in SEM media without ethanol. Total activities were measured with 1 mM ABTS in 100 mM sodium phosphate/citrate buffer, pH 4.0. Measurements were obtained from 8 independent microcultures in 96 well plates (microtiter fermentations) and expressed as the mean plus standard deviation.

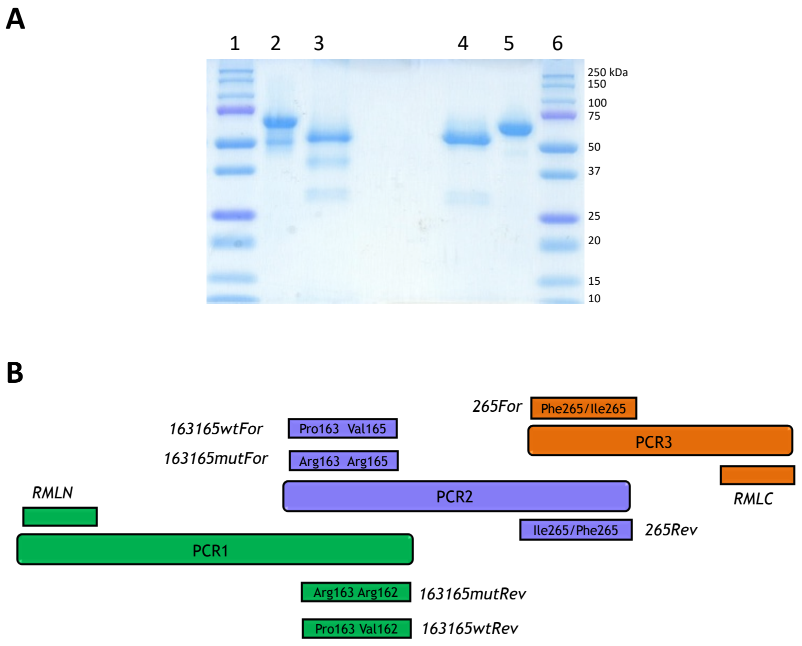

Figure S4. A) SDS-PAGE of purified LacAnc98 and LacAnc100. Lanes: 1, protein ladder; 2, LacAnc98; 3, deglycosylated LacAnc98; 4, deglycosylated LacAnc100; 5, LacAnc100; 6, protein ladder. The purified enzymes were desglycosylated using Peptide N-glycanase (PNGase F). Samples were resolved on 12% SDS-polyacrylamide gel and stained with ProtoBlue Safe. **B)** Site directed recombination *in vivo*. Primers designed for the PCR amplifications of the selected mutated positions (in black) in the site-directed recombination experiments to shuffle the best clones from saturation mutagenesis library.

A

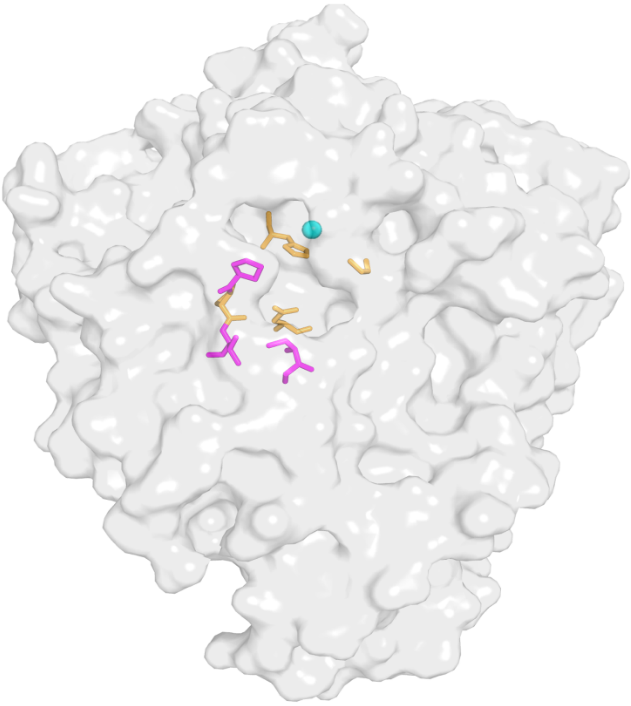

B

LacAcn100 **P163R/V165R**

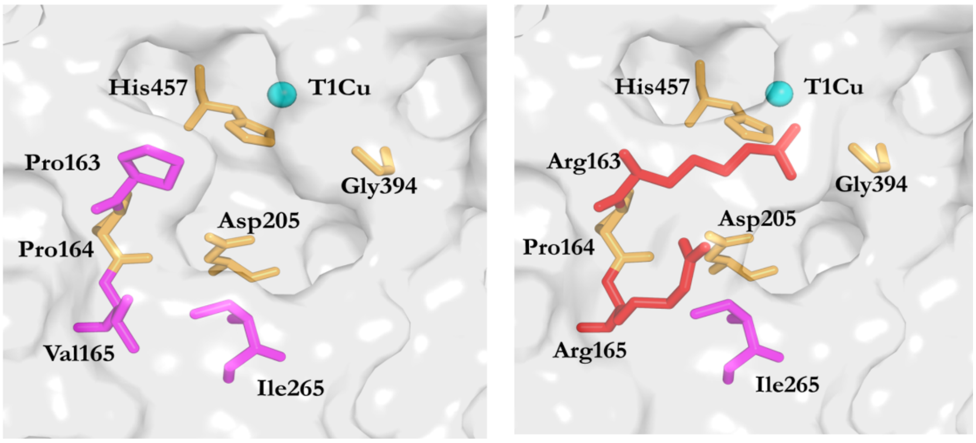

Figure S5. Molecular model of LacAnc100 laccase including mutations from saturation mutagenesis experiments. A) The LacAnc100 structure is shown as a light grey surface with some of the residues implicated in substrate interaction represented as sticks. **B)** Some of the residues of the catalytic pocket are depicted in orange while the selected residues for saturation mutagenesis are highlighted in magenta (left) and the substituted residues in clone 6 are shown in red (right). T1 copper is represented as a blue sphere. The model was made using Phyre2 server (Protein Homology/analogY Recognition Engine V 2.0) available at www.sbg.bio.ic.ac.uk/phyre2. The outcome model was used for modeling the mutants by Pymol (Schrodinger, LLC [http://www.pymol.org]).

Table S1. Laccases sequences used for ancestral reconstruction. GI numbers were accessed from GenBank.

GI number			
28268547	409151767	751715398	621090605
380704397	630209062	751711089	55670399
10801036	758348005	751681404	342161416
238632213	56785434	751672765	255523026
761951808	38194441	385282687	751000472
597970335	749841057	597933438	302672386
598003325	327349048	597933466	3273348
761926573	751056491	749880787	595781984
761926579	113207314	749885673	595781910
89274031	752354829	108936945	270047922
527300376	750965362	88687733	630355769
527300379	50724580	385141759	630350115
558633451	646305153	385139612	170101418
558633467	124495024	572695304	628856226
751689593	9957143	380750098	170101420
751689606	242220107	449546190	628856225
749897724	242210489	751007004	595781910
749897755	568441732	83415007	270047924
763724579	599119398	2833190	630359269
763725325	599096545	2833189	630350115
270485111	16041065	636611527	
348609404	34922426	46578391	
348609402	471887945	553303989	
409151775	471872355	553303985	
